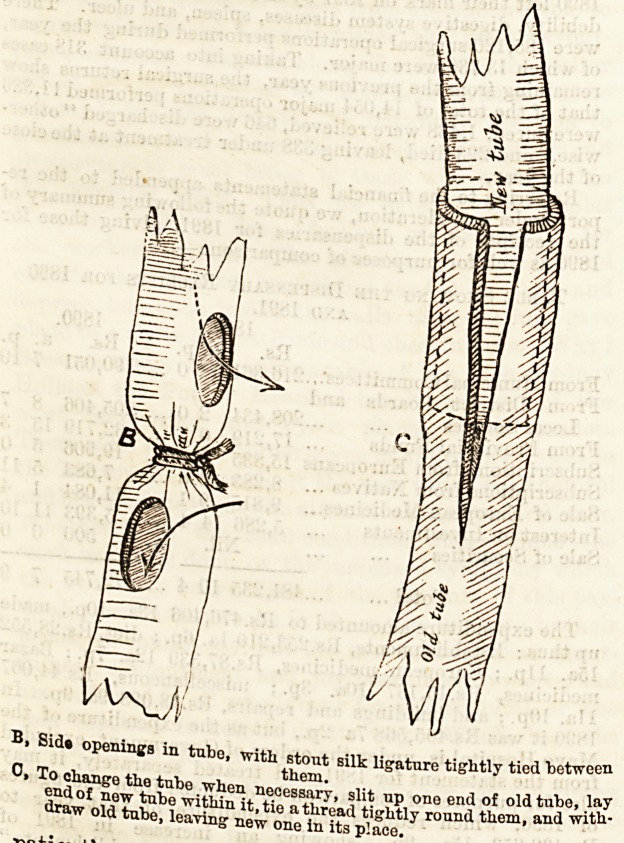# The Treatment of Empyema

**Published:** 1893-10-28

**Authors:** 


### LIVERPOOL ROYAL INFIRMARY.

### The Treatment of Empyema.

In a former article we described in detail the treatment of serous effusion in the pleura as carried out in the Royal Infirmary; we have now to describe the treatment adopted when the effusion is purulent.

As regards the diagnosis of this condition, its signs and symptoms are scarcely distinguishable from those of serous effusion, and though a persistent rise of temperature may lead us to suspect that pus is present, the diagnosis can only be established by the use of the aspirator or the exploring syringe.

Once pus has formed the possibility of absorption can hardly be entertained, and as in the case of an abscess elsewhere, early evacuation of the matter is demanded. This may conceivably be effected in two ways, either by aspiration, without the admission of air into the pleura, or by making a free incision, which must of necessity allow the air to enter the pleura and so favour collapse of the lung. It may safely be asserted that in adults aspiration alone will never lead to recovery, though as we shall see directly it may be a useful adjunct to the severer operation; but in children it is otherwise, and in them recovery

A, Diagram showing tube *in situ*.

sometimes follows one or more aspiration. Dr. A. T. H. Waters (*Contributions to Clinical and Practical Medicine*) states that the oldest patient in whom he has found it to succeed was a boy aged 7. Half a pint of pus was removed by aspiration, and five ounces more after an interval of four weeks. No further accumulation took place, and complete recovery followed.

Although in adults a free incision is inevitable, a preliminary aspiration is generally a useful proceeding; it gives immediate relief, removes pressure from the lung, and reduces the fever, and it can be done at once without waiting to prepare the patient for an anæsthetic. It may further be useful by encouraging the lung to expand, and even allowing it to become fixed in a position of expansion by the formation of pleural adhesions.

These principles of treatment are usually carried out in the following way: When the matter is first discovered, as much as will flow is drawn off in the ordinary way by the aspirator. After a few days have elapsed it is generally apparent that the matter has collected again, and the operation to establish free drainage is forthwith resorted to. An anæsthetic having been administered, the exact position of the matter is determined by means of the aspirator, and an opening is made through an intercostal space at this point. An attempt is then made by inserting the finger or a probe to ascertain the extent of the cavity, and a counter opening is made into it through an intercostal space at some distance behind and below the first incision. A stout drainage tube, as thick as the space will admit, is then passed through from one opening to the other, the ends being left protruding. This tube has been previously tied in the middle with a strong silk ligature, on either side of which side slits have been cut. It thus practically forms two independent tubes, and if syringing is required in the after treatment, the fluid must find its way into the pleural cavity. Any masses of lymph that present themselves are washed away, and an antiseptic dressing is applied.

Occasionally the ribs are so close together that there is no room to insert a tube between them. In such cases the periosteum is stripped off a rib for a short distance, and a piece of the bone removed. When this is done a single opening in the pleura is usually sufficient to insure complete drainage. The after treatment is simple, being chiefly directed to insuring a free discharge until the cavity closes, and maintaining the

B, Side openings in tube, with stout silk ligature tightly tied between them.
C, To change the tube when necessary, slit up one end of old tube, lay end of new tube within it, tie a thread tightly round them, and withdraw old tube, leaving new one in its place.

patient's strength. The chief difficulty is to keep the tube clear, and for this the plan adopted by Mr. R. A. Bickersteth is very useful. He leaves the projecting ends of the tube so long that when the wound is dressed one half can be drawn out of the chest without displacing the other half. In this way each segment of the tube can be in turn thoroughly cleansed up to the knot. After a few days a fresh tube is inserted in place of the old one. This is done in the way shown in Fig. C. A slit is made in the end of the old tube, and the end of the fresh tube slipped into its lumen, where it is fixed by a thread tied tightly round. As the old tube is drawn out the new one is drawn into position. When the cavity has diminished, a tube is retained only in the posterior opening, and the latter tube is replaced by a smaller one before the opening is allowed to close.

The results of this mode of treatment have been highly satisfactory. In many cases the subsequent deformity of the chest has been but slight, and in some the external cicatrices are the only remaining signs of the serious condition through which the patient has passed.

## Figures and Tables

**A f1:**
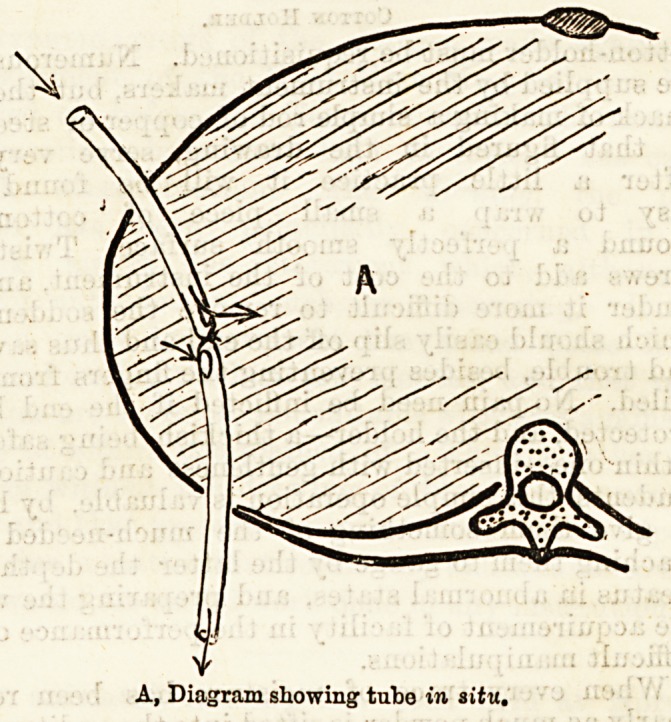


**B, C f2:**